# Overview of the development, characterization, and function of human types 1, 2, and 3 innate lymphoid cells

**DOI:** 10.31744/einstein_journal/2024RW1042

**Published:** 2024-11-06

**Authors:** Laiz Cameirão Bento, Nydia Strachman Bacal, Luciana Cavalheiro Marti

**Affiliations:** 1 Hospital Israelita Albert Einstein Clinical Pathology Laboratory São Paulo SP Brazil Clinical Pathology Laboratory, Hospital Israelita Albert Einstein, São Paulo, SP, Brazil.; 2 Hospital Israelita Albert Einstein Experimental Biology Laboratory Prof. Dr Geraldo Antonio de Medeiros Neto São Paulo SP Brazil Experimental Biology Laboratory Prof. Dr Geraldo Antonio de Medeiros Neto, Hospital Israelita Albert Einstein, São Paulo, SP, Brazil.

**Keywords:** Lymphoid progenitor cells, Intraepithelial lymphocytes, Lymphocytes, Cytokines, Immunity, innate lymphoid cells, Neoplasm

## Abstract

Hematopoiesis is characterized by the differentiation and maturation of multipotent stem cells into hematopoietic cells. Common lymphoid progenitor cells differentiate into B and T lymphocytes; natural killer cells can also originate from common lymphoid progenitors. In recent years, a cellular subtype of lymphocytes, called innate lymphocytes, has been described. Innate lymphoid cells (ILCs) play an important effector and regulatory role in innate immunity, and similar to natural killer cells, depend on the γc and Id2 chains for their development. These cells are divided into three main subtypes according to their characteristics, namely type 1 innate lymphocytes (ILC1), type 2 (ILC2), and type 3 (ILC3); the production of cytokines and transcription factors is essential for this classification. Furthermore, these cells have high plasticity, which allows them to change their phenotype in response to the environment. ILCs have recently been characterized further and emerged as a family of effectors and regulators of innate immune responses. Uncontrolled activation of these cells can contribute to inflammatory, autoimmune diseases and cancer. The current review aimed to describe their main characteristics, immunophenotypes, and plasticity, and based on the existing literature, suggested a phenotypic analysis to differentiate innate lymphocytes from natural killer cells, and across the subsets.

## INTRODUCTION

During hematopoiesis, the common lymphoid progenitors (CLPs) differentiate into precursors that are committed to cell lineages with antigen receptors, namely B and T cells.^(1)^ Cells without antigen receptors have also been described to originate from the CLPs; these include natural killer (NK) cells and lymphoid tissue inducers (LTi). In 1975, NK cells were first described as innate immune cells with cytotoxic effects.^(2)^ In 1997, LTi was described to be essential for lymph node formation during embryogenesis in mice.^(3,4)^ NK cells and LTi have related developmental processes, since both cell types require the common gamma chain (γc) of interleukin-2 (IL-2) receptor and the transcriptional repressor Id2 for their development.^(5,6)^

Recently, a new family of lymphocytes was described as innate lymphoid cells (ILCs).^(7,8)^ ILCs constitute a heterogeneous population of innate lymphocytes lacking gene rearrangement receptors; instead, they express germline-encoded inhibitory and activating receptors. Furthermore, they display important effector and regulatory functions in innate immune response, and similar to NK and LTi, depend on the gc and Id2 chains for their development.^(9,10)^

ILCs have been classified into three main groups according to their phenotypic characteristics, cytokine profiles, and the transcription factors necessary for their differentiation. The three groups are type 1 innate lymphocytes (ILC1), type 2 innate lymphocytes (ILC2), and type 3 innate lymphocytes (ILC3), which demonstrate distinct patterns of cytokine production resembling the pattern of cytokines secreted by CD4 T helper (Th)1, Th2, and Th17 lymphocyte subsets, respectively.^(1,9,11)^

Innate lymphocytes play an important role in the initial immune response to pathogens. They are innate immune cells that are part of the first line of defense against invading agents. The developed immunity provides defense against infectious agents, and the cells of innate and adaptive immunity not only participate in the immune response but are also involved in tissue repair, remodeling processes, and homeostasis preservation.^(12-14)^

ILCs have been characterized over the last 15 years and are evolving into effector and regulatory cells of innate immune response. However, human ILC characteristics and ontogeny are still emerging in the literature, and here, we have summarized the main data on them obtained in recent years.

### Type 1 innate lymphocytes (ILC1)

Some authors have classified type 1 innate lymphocytes into two subsets, comprising of NK cells and ILC1; however, the discussion remains controversial in the literature. ILC1 expresses the T-box transcription factor (T-bet), as observed in Th1 cells, and after exposure to interleukin (IL)-12, it is activated and can produce interferon-gama (IFN-γ).^(11,15)^

**Figure 1:**
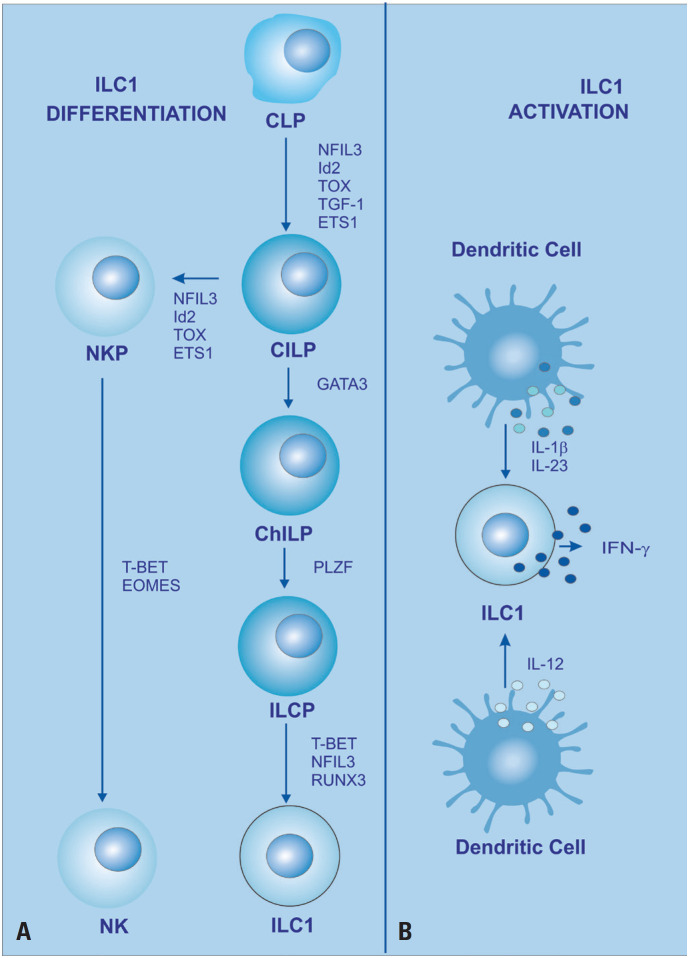
ILC1 development. (A) Schematic of the developmental pathway of ILCs. ILCs are believed to develop from common innate lymphoid progenitors (CILPs), which originate from common lymphoid progenitors (CLPs). CILPs can differentiate into NK cell precursors (NKPs) or common helper innate lymphoid progenitors (CHILPs), which in turn give rise to innate lymphoid cell precursors (ILCPs). ILCPs develop into ILC1, ILC2, or ILC3, respectively, the stage of differentiation being dependent on the expression of the indicated transcription factor. This pathway is mainly based on mouse ILC differentiation pathways;^(16)^ (B) ILC1 releases cytokines upon activation by dendritic cells^(20)^

Natural killer cells and ILC1 share several characteristics, such as cell surface markers, IFN-γ production, and the requirement of T-bet for their maturation. Despite the similarities, there are differences based on developmental pathways and functional distinctions.^(16)^ Both NK cells and ILC1 are believed to originate from CLPs, although CLPs can differentiate into two different precursors, a common ILC precursor (ILCP) that can produce all ILC subtypes, and an NK precursor (NKP) that can give rise to only NK cells^(17)^ (Figure 1A). The origin, however, is not well defined in the literature yet, since there is no reliable description of a specific precursor of ILCs, especially in humans.^(16,18)^

Eomes: eomesodermin; ETS1: avian erythroblastosis virus E26 homolog-1; GATA3: GATA-binding protein 3; NFIL3: nuclear factor IL-3 induced; Id2: inhibitor of DNA binding 2; PLZF: promyelocytic leukemia zinc finger; RUNX3: runt-relatedtranscription factor 3; T-bet: T-box transcription factor; TOX: thymocyte selection-associated high-mobility group box protein.

Furthermore, ILC1 does not express killer cell immunoglobulin-like receptor 3 Ig domains and long cytoplasmic tail 1 (KIR3DL1) or interleukin 15 receptor (IL15R), which is essential for NK cell development.^(16-18)^

Natural killer cells play a cytolytic role and migrate through blood vessels to different regions of the body, whereas most ILC1 cells are tissue-resident and do not have cytolytic activity. Quite frequently, studies have demonstrated that ILC1 differs from NK cells due to the lack of expression of CD16, CD56, CD94, perforin, or granzyme B.^(18,19)^ ILC1 does not express CD117 (c-kit) or NKp44, but highly expresses CD127 (IL-7Rα), which distinguishes it from immature NK cells.^(19)^ The ILC1 phenotype includes the expression of CD127, CD161, T-bet, and IFN-g, and excludes the expression of Eomes, CD56, NKp44, KIR, and Perforin.^(20)^ However, such characteristics are further complicated by reports of different ILC1 subsets with heterogeneous expression of transcription factors, such as T-bet and Eomes. One such example is intraepithelial ILC1, which lacks CD127 expression but expresses CD103 and NKp44.^(20,21)^However, according to several authors, ILCs are not NK cells or T cells, since they are distinct from conventional NK cells, T cells, MAIT cells, and CD1- restricted T cells, such as NKT cells.^(18,20,22)^

ILC1 is activated by intracellular bacteria, viruses, and some parasites, regulates tissue homeostasis, and displays antitumor properties.^(21)^ It assumes a dominant role in the adult intestine since the upper gastrointestinal tract, such as the esophagus, is enriched in it. Its frequency is higher in the inflamed intestines of patients with Crohn's disease, indicating its important role in the pathogenesis of intestinal mucosal inflammation.^(22,23)^ The significance of ILCs in intestinal infection has been demonstrated in various experimental models, since ILC1 can control intracellular pathogens and viruses through IFN-g production.^(22)^

ILC1 has also been reported to be present in the human decidua during the first trimester of pregnancy, suggesting its involvement in the inflammatory phase of early pregnancy, mainly for embryo implantation.^(24)^ In peripheral blood, ILC1 was described as expressing CD127 and CD161 while lacking lineage, CD56, CD94, CD117, and CRTH2 expression.^(25)^

In the tissue microenvironment, cells produce cytokines in response to infection, inflammation, and tissue injury. ILC1 is activated by interleukin (IL)-12 and is responsible for releasing IFN-γ and tumor necrosis factor-alpha (TNF-α) (Figure 1B).^(16,26,27)^

The ILC1 immunophenotype remains poorly described in the literature, and no specific marker has defined the population yet. Several markers expressed by NK cells are shared by ILC1, making their characterization even more difficult. Furthermore, ILC1 characteristics may vary depending on the tissue analyzed.^(16,27)^ Currently, ILC1 is described in mucosa-associated lymphoid tissues (MALT), such as the intestine (GALT), tonsils (O-MALT), lungs (BALT), skin (SALT), and others such as the liver, endometrium, uterine decidua, and peripheral blood.^(27-30)^

**Figure 2:**
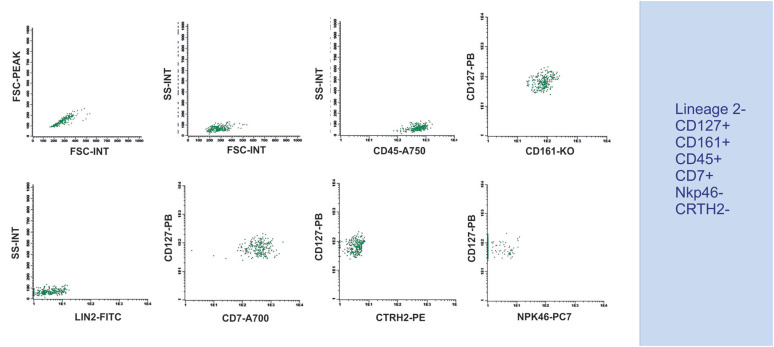
Strategy for ILC1 identification in human peripheral blood - Flow cytometry plots showing an initial gating in FSC-A *versus* FSC-H, followed by FSC-A *versus* SSC-A, CD45-positive cells, double-positive staining for CD127 and CD161, negative staining for Lineage 2 (Lin2), positive staining for CD7, and negative staining for CRTH2 and NKP46

In several other tissues, including the human bone marrow, ILC1 has not yet been fully characterized or identified.^(18,30)^ Our team used some of the markers proposed in the literature (Table 1) to perform an analysis of human peripheral blood, for which we provided a suggested gating strategy and markers. The results showed a population that was lineage 2- (CD3, CD14, CD19, CD20, and CD56), CRTH2-, and NKp46-negative, and CD45-, CD127-, CD161-, and CD7-positive (Figure 2). The major distinguishing factor between ILC1 and NK cells was CD56. The essential distinctions across the ILCs were CTRH2 and NKp46 expression; when ILC2 was positive for CTRH2, ILC1 and ILC3 were negative, and ILC1 and ILC2 did not express NKp46, although ILC3 did.

**Table 1:** The main immunophenotypic markers of ILCs described in the literature^(16)^

	Subset of human innate lymphocyte					
	Marker	NK	ILC1	ILC 2	ILC3 NKp44^-^	ILC3 NKp44^+^
Cell surface markers	CD45	+	+	++	+	+
	CD127 (IL-7Ra)	+/-	+	+	+	+
	CD161 (NK1.1)	+/-	+	+	+	+
	ST2 (IL-33R)	+/-	-	+/-	*	-
	CD276 (ICOS)	-	*	+	*	+
	IL-17RB (IL25R)	-	-	+	*	-
	CD294 (CRTH2)	-	-	+	-	-
	KLRG1	+	-	+	-	-
	CD117 (c-kit)	-/w	-	+/-	+	+
	CD69	w	+/-	*	*	*
	CD254 (RANKL)	-	*	*	+	+
	CD196 (CCR6)	-	+/-	+/-	+/-	+/-
	CD335 (NKp46)	+	-	-	+/w	+/w
	CD25 (IL-2Ra)	+/-	w	+	+/-	-/w
	MHC-II	+/-	*	+/-	*	+/-
	IL23R	+/-	+/-	-/w	+	+
	IL1R	+/-	+	w	+	+
	CD122	+	*	*	w	w
	CD314 (NKG2D)	+	*	*	-/w	-/w
	KIR	+/-	-	-	-	-
	CD94	+/-	-	-	-	-
	Perforin	+	-	-	-	-
	IL-12RB	+	+	-	-	+/-
	CD194 (CCR4)	*	+/-	+	*	*
	CD56	+	-	-	+/-	+/-
	CD183 (CXCR3)	*	+	*	*	*
	CD337 (NKp30)	+	+	+	+/-	+/-
	CD336 (NKp44)	+/-	-	-	-	+
	CD16	+/-	-	-	-	-
	NKp80	+	-	*	*	*
Transcription factors	Tbet	+	+	-	-	-
	Eomes	+	+/-	-	-	-
	RORgt	-	-/w	-/w	+	+
	GATA3	-/w	-/w	+	-/w	-/w
	AhR	- w	-/w	+	+	+

* Not determined,

+/- expression is detected in some but not in all cells,

-/weak no expression or weak expression,

w weak expression.

Lineage 2: CD3, CD14, CD19, CD20, CD56.

The literature compiled for describing ILC1 is listed in table 2.

**Table 2:** Main articles related to ILC1 characteristics

References	Journal	Study subject	Study type
Hazenberg et al., 2014^(11)^	Blood	General overview on ILCs	Review article
Juelke et al., 2016^(15)^	Curr Opin Immunol	Overview in ILC lineage commitment	Review article
Vivier et al., 2018^(16)^	Cell	Overview on ILC differentiation and characterization	Review article
Renoux et al., 2015^(17)^	Immunity	NK progenitor cell differs from ILC progenitor	Original article
Roan et al., 2017^(18)^	Immunity	Specifically discuss ILC1 markers expression	Letter
Bernink et al., 2013^(19)^	Nat Immunol	ILC1 in inflamed mucosal tissues	Original article
Krabbendam et al., 2018^(20)^	Curr Protoc Immunol	ILCs isolation protocol	Original article
Fuchs et al., 2013^(21)^	Immunity	ILC1 is responsive to IL-12 and IL-15	Original article
Panda et al., 2019^(22)^	Front Immunol	Mucosal ILCs	Review article
Zheng et al., 2022^(23)^	Int J Mol Sci	ILCs in Intestinal inflammatory disorders	Review article
Vacca et al., 2015^(24)^	Mucosal Immunol	ILCs in human decidua	Original article
Bennstein et al., 2019^(25)^	Cytometry A	Blood and neonatal ILCs characterization	Original article
Ebbo et al., 2017^(26)^	Nat Rev Immunol	ILCs in inflammatory diseases	Review article
Colonna, 2018^(27)^	Immunity	Diversity of ILCs diversity and redundant functions	Review article
Gasteiger et al., 2015^(28)^	Science	Tissue resident ILCs	Original article
Simoni et al., 2018^(29)^	Immunology	Heterogeneity of human ILC	Review article
Mjösberg et al., 2016^(30)^	J Allergy Clin Immunol	Human ILCs	Review article

### Type 2 innate lymphocytes (ILC2)

ILC2 have been described as cells with lymphoid morphology and absence of markers for T, B, or myeloid lineage. In humans, these cells have been reported to be present in several organs and tissues, such as the gut, lungs, tonsils, adipose and nasal tissues, skin, and peripheral blood.^(1,15,31)^ In 2011, some cells were initially described as an immature NK subset, functionally characterized by low/absent cytotoxicity, and lacking granzyme, perforin, and killer immunoglobulin receptors (KIR); they were later identified as ILCs.^(32)^

**Figure 3:**
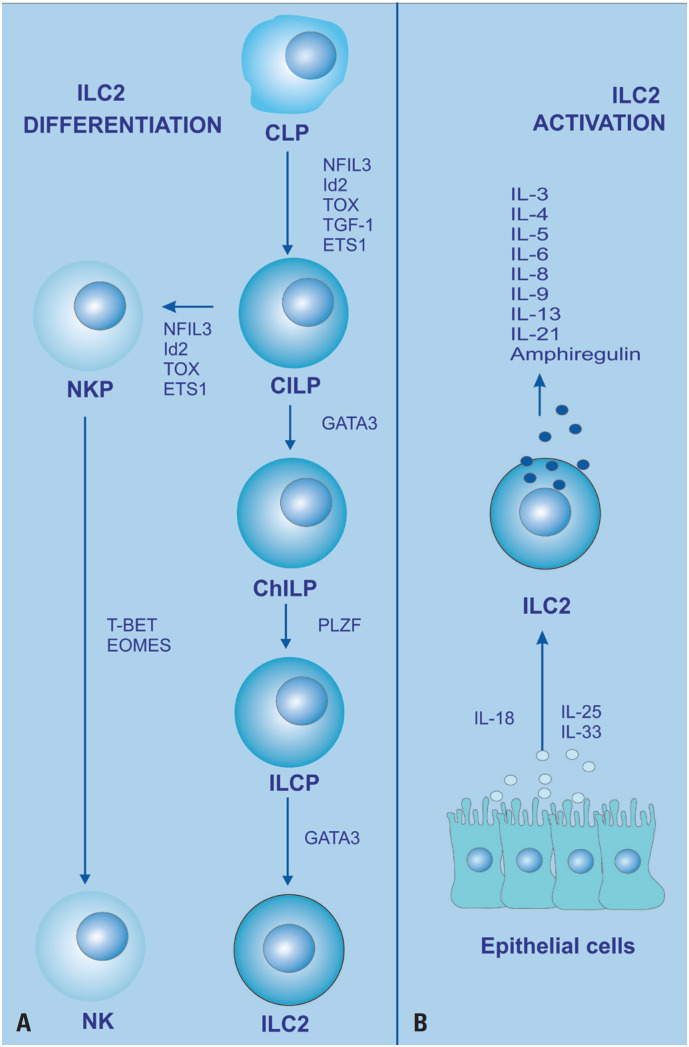
ILC2 development. (A) The developmental pathway of ILCs is illustrated. ILCs are thought to develop from common innate lymphoid progenitors (CILPs), which are derived from common lymphoid progenitors (CLPs). CILPs can differentiate into NK cell precursors (NKPs) or into common helper innate lymphoid progenitors (CHILPs), which subsequently give rise to innate lymphoid cell precursors (ILCPs). ILCP develops into ILC1, ILC2, or ILC3, respectively, the stage of differentiation being dependent on the expression of the indicated transcription factor. The pathway is mainly based on mouse ILC differentiation pathway;^(16)^ (B) ILC2 releases cytokines upon activation by epithelial cell alarmins^(19,32)^

ILC2 expresses the transcription factor GATA3 and produces interleukins (Th2-related), such as IL-4, IL-5, and IL-13, in response to stimulation by tissue alarmins IL-25, IL-33, and thymic stromal lymphopoietin (TSLP), which were initially identified as non-T and non-B cell sources of type 2 cytokines (Figure 3).^(19,33-35)^ Indeed, some ILC2 subsets express low levels of IL-33, IL-25 (IL-17E), and TSLP receptors; they are predominantly activated by IL-18, which is highly expressed in ILC2 from the skin, lungs, and bone marrow.^(34,35)^

Eomes: eomesodermin; ETS1: avian erythroblastosis virus E26 homolog-1; GATA3: GATA binding protein 3; NFIL3: nuclear factor IL-3 induced; Id2: inhibitor of DNA binding 2; PLZF: promyelocytic leukemia zinc finger; RUNX3: runt-related transcription factor 3; T-bet: T-box transcription factor; TCF-1: T cell factor 1; TOX: thymocyte selection-associated high-mobility group box protein.

Production of growth factors, such as granulocyte-macrophage colony-stimulating factor (GM-CSF) and amphiregulin, and interleukins, such as IL-3, IL-6,IL-8, IL-9, and IL-21, by ILC2 has already been reported earlier. All these molecules play an important role in the innate immune response against allergens, helminths, and nematodes and are involved in metabolic homeostasis and tissue repair.^(31)^

The main physiological role of ILC2 is in allergic processes and parasitic infections. However, little is known about the role of ILC2 in human parasitic infections.^(31)^

In mice, ILC2 are generated from a common lymphoid progenitor (CLP), common innate lymphocyte progenitor (CILP), and common helper innate lymphocyte progenitor (CHILP) (Figure 3), whereas NK cell-restricted progenitors (NKP) can give rise to only NK cells.^(17)^ However, in humans, this type of precursor has not been identified yet, and the source of human ILC2 remains unclear.

The identification of ILC2 in human thymus and the capacity of CD34-positive and CD1a-negative thymocytes differentiating into ILC2 *in vitro* under the influence of a delta-notch ligand had previously been reported. However, the study did not demonstrate the role of thymus in the development of ILC2, only indicating the presence of progenitors with the capacity to differentiate into these cells in the thymus.^(36)^

ILC2 are enriched in adipose tissue and were initially identified in murine white adipose tissue (WAT)-associated lymphoid clusters. IL-33 administration resulted in elevated numbers of ILC2 in WAT, which was associated with an increase in beige adipocytes, improved glucose tolerance, and greater weight loss, suggesting that ILC2 can interact with other cell types within adipose tissue and support metabolic homeostasis.^(37)^

In the lungs, activated ILC2 can secrete large amounts of type 2 cytokines, such as IL-5, IL-9, IL-13,and amphiregulin. These cytokines interfere with different tissue areas and promote cell recruitment, tissue remodeling, and cell differentiation.^(38)^ IL-5, when secreted by ILC2, promotes eosinophil recruitment to the site of inflammation. IL-13 leads to the hyperplasia of goblet cells in the lungs, causing mucus hyperproduction. During tissue repair, IL-13 promotes fibrosis by stimulating fibroblasts and macrophages, contributing to the pathogenesis of asthma and other lung diseases. The secretion of IL-13 by ILC2 further facilitates the differentiation of helper T cells into Th2 cells, leading to an even more accentuated inflammatory process.^(38,39)^At the beginning of inflammation, ILC2 transiently produces IL-9, which promotes mast cell accumulation at the inflammatory sites and airway remodeling. Furthermore, it amplifies the production of IL-5 and IL-13, promoting the survival and/or activation of ILC2 in an autocrine/paracrine manner and generating pro-inflammatory effects. Once activated and proliferating, majority of the activated lung ILC2 remain in the lung and presumably die when their function is completed, although the activation-induced death of ILC2 has not been studied yet. A small proportion of the activated lung ILC2 persists in the lungs as memory-like ILC2s or becomes exhausted.^(38-40)^

ILC2s have a high capacity to produce Th2-like immune responses after epithelial injury, and are consequently associated with several airway diseases, including asthma, pulmonary fibrosis, chronic rhinosinusitis with nasal polyps, allergic rhinitis, respiratory infections caused by influenza, and respiratory syncytial viruses. However, amphiregulin production by ILC2 benefits the repair of damaged airway epithelium and restores any lung function impaired by viral infection.^(41-43)^

A few studies have shown that ILC2 are present in allergic skin inflammation, such as atopic dermatitis. Epithelial lesions in this disease show high expression of the cytokines IL-25 and IL-33, which can induce ILC2 to secrete IL-4 and IL-13.^(38)^

ILCs have been described in the fetal intestine, in the intestines of healthy individuals, and in patients with intestinal inflammation. ILC2 were originally identified in the gastrointestinal tissues and fat-associated lymphoid clusters, highlighting the important role of these cells in mediating protective immuneresponses and inflammation in the gut. IL-25 and IL-33-responsive ILC2 are critical for IL-13 secretion and intestinal goblet cell hyperplasia, facilitating the expulsion of worms in the absence of adaptive immunity. However, the accumulation of these cells during intestinal inflammation in humans still needs to be clarified.^(43)^

ILC2 plays an important role in the immune responses to inflammatory and allergic diseases in the epithelial tissue and respiratory tract, gradually revealing an important role in the regulation of the immune system and tissue homeostasis.^(41-43)^ Albeit ILC2s are not found in many organs or tissues, only few tissues, including human bone marrow, have been investigated for the presence of ILC2.

Many research groups have defined ILC2 to be positive for CD25, CD127, CD161, and CRTH2, although these markers vary depending on the tissue analyzed.^(11,15,16,39,43)^

**Figure 4:**
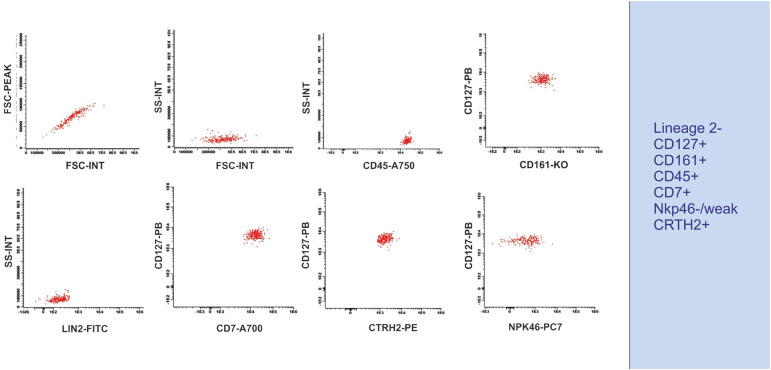
Strategy for ILC2 identification in human peripheral blood - Flow cytometry plots showing an initial gating in FSC-A *versus* FSC-H, followed by FSC-A *versus* SSC-A, using CD45-positive cells, double-positive staining for CD127 and CD161, negative staining for Lineage 2 (Lin2), positive staining for CD7 and CRTH2, and negative/weak staining for NKP46

Our team used some of the markers proposed in the literature (Table 1) to perform an analysis of human peripheral blood, for which we have provided a suggested gating strategy and markers. The results revealed a population that was lineage 2 (CD3, CD14, CD19, CD20, and CD56)-negative, CRTH2-positive, NKp46-negative, and CD45-positive, CD127-positive, CD161-positive, and CD7-positive (Figure 4).

Lineage 2 (CD3, CD14, CD19, CD20, CD56).

The literature used for describing ILC2 is listed in table 3.

**Table 3:** Main articles related to ILC2 characteristics

References	Journal	Study subject	Study type
Artis et al., 2015^(1)^	Nature	ILCs biology	Review article
Hazenberg et al.,2014^(11)^	Blood	General overview on ILCs	Review article
Juelke et al., 2016^(15)^	Curr Opin Immunol	Overview in ILC lineage commitment	Review article
Vivier et al., 2018^(16)^	Cell	Overview on ILC differentiation and characterization	Review article
Renoux et al., 2015^(17)^	Immunity	NK progenitor cell differs from ILC progenitor	Original article
Krabbendam et al., 2018^(20)^	Curr Protocol Immunol	ILCs isolation protocol	Original article
Moro et al., 2010^(31)^	Nature	Description of ILC2	Original article
Tang et al., 2011^(32)^	Blood	ILC2 described as NK subset	Original article
Weston et al., 2019^(33)^	J Allergy Clin Immunol	Functional chemokine receptors in ILC2	Original article
Ricardo-Gonzalez et al., 2018^(34)^	Nat Immunol	ILC2 identity according to tissue origin	Original article
Spits et al., 2013^(35)^	Nat Rev Immunol	ILCs uniform nomenclature	Review article
Gentek et al., 2013^(36)^	Front Immunol	ILC2 induction by NOTCH signal modulation	Original article
Schuijs et al., 2019^(37)^	Ann N Y Acad Sci	ILC2 in innate and adaptive immunity	Review article
Mathä et al., 2021^(38)^	Front Immunol	ILC2 fate	Review article
Turner et al., 2013^(39)^	J Exp Med	ILC2 survival induced by IL-9 and lung protection	Original article
Halim, 2016^(40)^	Int Immunol	ILC2 in disease	Review article
Kim et al., 2015^(41)^	Cold Spring Harb Perspect Biol	ILC2 in health and disease	Review article
Peebles, 2015^(42)^	J Leukoc Biol	ILC2 in human disease	Review article
Meininger et al., 2020^(43)^	Trends Immunol	ILCs phenotype	Review article

### Type 3 innate lymphocytes (ILC3)

In humans, ILC3 have been described in tissues, such as the tonsils, intestine, lymph nodes, spleen, skin, and peripheral blood; however, the ontogeny of these cells is not yet well known.^(16,17)^ Few *in-vitro* studies have demonstrated the possible origin of ILC3 from a progenitor present in human tonsils, spleen, and lymph nodes.^(44-46)^ Furthermore, a previous study had demonstrated the capacity of innate lymphocytes to differentiate from progenitor cells derived from the peripheral and umbilical cord blood. However, owing to the lack of studies on normal bone marrow, the path of maturation of ILC3 *in vivo* was not known until our data were obtained.^(47)^

**Figure 5:**
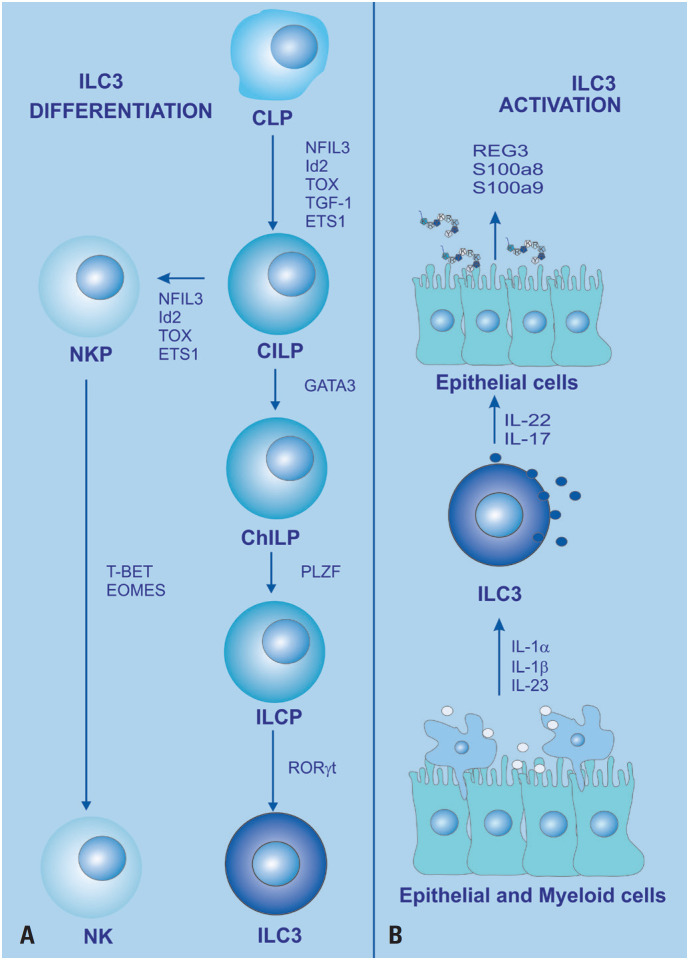
ILC3 development. (A) Schematic for the developmental pathway of ILCs. ILCs appear to be derived from common innate lymphoid progenitors (CILPs), which in turn originate from common lymphoid progenitors (CLPs). CILPs can differentiate into NK cell precursors (NKP) or common helper innate lymphoid progenitors (CHILPs), which give rise to innate lymphoid cell precursors (ILCPs). ILCP will develop into ILC1, ILC2, or ILC3, respectively, the stage of differentiation being dependent on the expression of the indicated transcription factor. The pathway is mainly based on mouse ILC differentiation pathways;^(16)^ (B) ILC3 releases cytokines upon activation by cytokines secreted from epithelial cells and myeloid cells^(50,51)^

ILC3 plays an important role in intestinal tissue homeostasis and is typically activated by IL-1b and IL-23. This subtype is responsible for the production of IL-22, a cytokine that acts on intestinal epithelial cells. IL-22 induces a cascade of gene expression that leads to the secretion of antimicrobial peptides, such as REG3, S100a8, and S100a9 (Figure 5).^(48,49)^

Eomes: eomesodermin; ETS1: avian erythroblastosis virus E26 homolog-1; GATA3: GATA binding protein 3; NFIL3: nuclear factor IL-3 induced; Id2: inhibitor of DNA binding 2; PLZF: promyelocytic leukemia zinc finger; RUNX3: runt-related transcription factor 3; T-bet: T-box transcription factor; TCF-1: T cell factor 1; TOX: thymocyte selection-associated high-mobility group box protein.

The production of cytokines by ILC3 regulates the function of the epithelial barrier with commensal bacteria in the intestine. Furthermore, these cytokines interact with the adaptive immune cells, promoting an intestinal microenvironment of tolerance and preventing inflammation caused by commensal bacteria.^(49,50)^On the other hand, ILC3 contributes to local tissue immunity and inflammation through the production of other cytokines, such as IL-17.^(33,51-53)^ Studies have shown that ILCs producing IL-17 cytokine may be associated with the pathogenesis of psoriasis, airway inflammation, and obesity.^(54-56)^ Moreover, patients with Crohn's disease have increased frequency of ILC3 (NKp44− and CCR6+) and elevated levels of IL-17.^(57)^

The production of proinflammatory cytokines, such as IFN-g and TNF-α, can as well contribute to immunity. However, chronic activation of ILC3 can result in diseases due to uncontrolled inflammation. Thus, in contrast to their protective and homeostatic roles, ILC3 can play pro-inflammatory roles and contribute to tissue damage and inflammation during disease.^(50)^

ILC3 can be classified according to NKp44+ and NKp44- expression. Depending on the stimulus, it can produce TNF-α, IL-17, IL-22, and GM-CSF, promoting antibacterial and anti-inflammatory responses or participating in tissue repair.^(1,57,58)^

IL-17 is typically produced by NKp44- cells, whereas IL-22 is produced by NKp44+ cells. This subtype expresses the receptor of retinoic acid γt (RORγt).^(59)^ Furthermore, the cells positively express CD7, CD127, CD117, CD161, and CCR6 while showing no expression of CD94 and CRTH2.^(11)^

**Figure 6:**
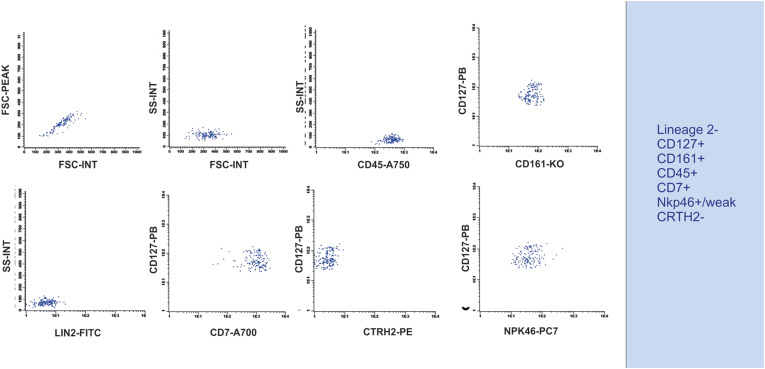
Strategy for identifying ILC3 in human peripheral blood - Flow cytometry plots showing an initial gating in FSC-A *versus* FSC-H, followed by FSC-A *versus* SSC-A, using CD45-positive cells, double-positive staining for CD127 and CD161, negative staining for Lineage 2 (Lin2), positive staining for CD7, and CRTH2 and positive/weak staining for NKp46

Our team used some of the markers proposed in the literature, as displayed in table 1, to perform an analysis of human peripheral blood, for which we provided a suggested gating strategy and markers. The results revealed a population that was negative for lineage 2 (CD3, CD14, CD19, CD20, and CD56), negative for CRTH2, positive/weak for NKp46, and positive for CD45, CD127, CD161, and CD7(Figure 6). The literature used for describing ILC3 is listed in table 4.

Lineage 2 (CD3, CD14, CD19, CD20, CD56).

**Table 4:** Main articles related to ILC3 characteristics

References	Journal	Study subject	Study type
Artis et al., 2015^(1)^	Nature	ILCs biology	Review article
Hazenberg et al., 2014^(11)^	Blood	General overview on ILCs	Review article
Vivier et al., 2018^(16)^	Cell	Overview on ILC differentiation and characterization	Review article
Renoux et al., 2015^(17)^	Immunity	NK progenitor cell differs from ILC progenitor	Original article
Spits et al., 2013^(33)^	J Allergy Clin Immunol	Functional chemokine receptors in ILC2	Original article
Chen et al., 2018^(44)^	Immunity	ILC3 developmental pathway	Original article
Freud et al., 2006^(45)^	J Exp Med	NK developmental pathway	Original article
Scoville et al., 2016^(46)^	Immunity	ILC progenitor	Original article
Moretta et al., 2016^(47)^	Eur J Immunol	Hematopoietic stem cell source influences ILC differentiation	Original article
Sonnenberg et al., 2011^(48)^	Nat Immunol	IL-22 and regulation of immunity	Review article
Mukherjee et al., 2015^(49)^	Immunity	Intestinal antimicrobial defense	Review article
Melo-Gonzalez et al., 2017^(50)^	Immunology	ILC3 phenotypic heterogeneity	Review article
Klose et al., 2016^(51)^	Nat Immunol	ILC in immune regulation	Review article
Walker et al., 2013^(52)^	Nat Rev Immunol	General overview on ILCs	Review article
Diefenbach et al., 2014^(53)^	Immunity	ILC development and function	Review article
Kim et al., 2014^(54)^	Nat Med	ILC3 and inflammasome	Original article
Villanova et al., 2014^(55)^	J Invest Dermatol	ILC3 in psoriasis	Original article
Dyring-Andersen et al., 2014^(56)^	Br J Dermatol	ILC3 in non-lesional psoriatic skin	Original article
Geremia et al., 2011^(57)^	J Exp Med	ILC3 in bowel disease	Original article
Cupedo et al., 2009^(58)^	Nat Immunol	ILC3 precursor cell	Original article
Montaldo et al., 2014^(59)^	Immunity	ILC3 precursor cell	Original article

### ILC plasticity

Plasticity is defined as the ability of an innate lymphocyte to acquire characteristics associated with another innate lymphocyte. This suggests the efficiency of the immune system in maintaining adequate conditions for tissue homeostasis without the need to recruit new cells.^(60)^

Few studies have demonstrated the ability of ILC2 to acquire the characteristics of ILC1 in inflammatory conditions, such as Crohn's disease and chronic obstructive pulmonary disease.^(61-64)^

This change occurs when ILC2 cells are exposed to IL-12 and lack GATA3, IL-5, and IL-13 expression, and start expressing T-bet and IFN-g-, transforming themselves into ILC1. However, when exposed to IL-4, the cells acquire the characteristic markers of ILC2.^(61,64)^

In addition, Bernink et al. demonstrated that ILC3 in humans can differentiate into ILC1 in the presence of IL-12. During this process, ILC3 loses the RORgt transcription factors and begins to express T-bet and IFN-g-, losing the ability to secrete IL-22, and becomes ILC1.^(65)^ This conversion is reversible when ILC1 is exposed to IL-1b, IL-2, and IL-23 and returns to expressing the characteristic markers of ILC3.^(65)^

## CONCLUSION

ILC1, ILC2, and ILC3 are three distinct subsets of cells in the innate immune system that play important roles in immune response. Each type of ILC has unique characteristics and specialized functions for defending the organisms against pathogens and maintaining homeostasis. Currently, the phenotype and function of ILCs have been identified; however, a more profound analysis of their phenotype and function in homeostatic and pathophysiological processes would be required for complete elucidation. They are important components of innate immunity and play fundamental roles in defending the organism against different types of pathogens and regulating the immune response. Understanding these cells and their specific functions can contribute to a more complete and detailed understanding of the functions of the immune system, paving the way for the development of new therapeutic approaches and strategies to combat diseases.
